# Supplementary material for: Adversarial attacks against supervised machine learning based network intrusion detection systems
Source: PLoS One. 2022 Oct 14;17(10):e0275971. doi: 10.1371/journal.pone.0275971 (PMC9565394; doi:10.1371/journal.pone.0275971)
Supplement: Attachment — Submitted filename: Response to Reviewers.pdf [file pone.0275971.s001.pdf]

Reply to Reviewers' Comments

Manuscript ID: PONE-D-22-13930
Title of Paper: Adversarial Attacks Against Supervised Machine Learning Based Network Intrusion Detection Systems.
Authors: Ebtihaj Saeed Alshahrani [1], Daniyal Alghazzawi[1], Reem Alotaibi[2], Osama Rabie[1]

1 *Information Systems Department, Faculty of Computing and Information Technology, King Abdulaziz University, Jeddah, Saudi Arabia*

2 *Information Technology Department, Faculty of Computing and Information Technology, King Abdulaziz University, Jeddah, Saudi Arabia*

* *correspondingauthor: ealshahrany0001@stu.kau.edu.sa*

We sincerely thank the editors to handle the paper and thank all the reviewers for providing valuable feedback to help us improve the quality of this paper. We have carefully considered all the review comments and revised the paper accordingly to address or clarify all the issues raised in the reviews. The following is a list of item-by-item responses to the review's comments.

[Response to Reviewer 1's Comments]

Comment 1: Abstract: section needs to be re-drafted to be self-contained means it has to clearly show the hypothesis, methodology, techniques and tools used, and the results obtained.

Response: Thank you for carefully reading the manuscript. The abstract has been edited to include all mentioned component in your comment.

Comment 2: Keywords: Authors suggested to update the keywords by selecting more relevant terms. Keywords play important role in the appearance of the manuscript in scholars search which will give it more hits and more citations.

Response: Thank you for carefully reading the manuscript. The word "adversary" has been added to the list of keywords. Additionally, we chose our research's keywords based on NIST standards, which are terms that are more relevant to our field.

Comment 3: Introduction: authors advised to add one more paragraph at the end of the section to show the organization of the rest of the paper.

Response: Thank you for your comment. We have updated the introduction and added the final paragraph to show the organization of the rest of the paper.

Comment 4: Authors suggested to go through the following references, and they MAY make use of them in updating the introduction and the related work sections:

Response: Thank you for your suggestion. The first study mentioned, which is more relevant to our study, is cited in the background section.

Comment 5: Conclusion: The conclusion should be abstracted so authors need to consider re-drafting it.
Response: **Thank you for carefully reading the manuscript. Based on your comment, we have abstracted the conclusion to be more clearly .**

Comment 6: Authors need to confirm that all acronyms are defined before being used for first time. Authors need to confirm that all mathematical notations are defined when being used for first time.
Response: Thank you for your comments. We have addressed the comments accordingly.

Comment 7: Authors suggested to proofread the manuscript after addressing all comments to avoid any typo, grammatical, and lingual mistakes and errors.
Response: We appreciate you taking the time to read the manuscript. The paper was meticulously checked and proofread to make sure the language is improved.

[Response to Reviewer 2's Comments]

Comment 1: The article is very well written with a robust methodology. I have a few points to add. First, in section 4, the libraries Pandas, Numpy, Matplotlib, and Sklearn must be cited correctly.

Response: We appreciate you taking the time to read the manuscript and give your comments. We added the references for the following libraries: Pandas, Numpy, Matplotlib, and Sklearn as you mentioned.

Comment 2: The conclusion section needs to be expanded, pointing out what the other publications mentioned in section 3 could not achieve. Also, the authors could highlight their results and contribution to the literature.

Response: Thank you for your suggestions. We have updated the conclusion according to your comments. Moreover, we have highlighted the contributions at the end of literature.

Thank you very much for reviewing our article.

Thank you again for your precise time and valuable efforts spent in reviewing our manuscript as well as your helpful and positive comments.

Sincerely,
The Authors
